# Bacterial strain type and TEM-1 enzyme allele impact antibiotic susceptibility distribution in monoclonal populations: a single-cell droplet approach

**DOI:** 10.1098/rsos.242143

**Published:** 2025-07-30

**Authors:** Shahab Shahryari, Shakeel Ahmad, Ilona Paulina Foik, Paweł Jankowski, Adam Samborski, Marcin Równicki, Shreyas Kandhadai Vasantham, Piotr Garstecki

**Affiliations:** ^1^ Department of Soft Condensed Matter, Microfluidics and Complex Fluids Research Group, Institute of Physical Chemistry, Polish Academy of Sciences, Warsaw, Poland; ^2^ Department of Immunology and Microbiology, Costerton Biofilm Center, University of Copenhagen, Copenhagen, Denmark; ^3^ Department of Pharmaceutical Biology, Microbiota Lab, Medical University of Warsaw, Warsaw, Poland

**Keywords:** microfluidics, clonal heteroresistance, antibiotic resistance, TEM-1 beta-lactamase

## Abstract

Bacterial populations can display different susceptibilities to antibiotics among individual cells, even though they originate from the same parent cell. This variability can lead to treatment failure and the emergence of resistant bacteria. Understanding the factors influencing this variability is crucial for developing effective antibiotic treatments. The underlying cause of variation in susceptibility distribution within bacterial populations remains unclear, necessitating the development of new tools for measurement. Here, we use a droplet microfluidic single-cell antibiotic susceptibility assay and focus on antibiotic resistance conveyed by the TEM β-lactamases family. We investigate how the catalytic activity of β-lactamase, and the genetic characteristics of the host strains affect the susceptibility distribution within bacterial populations at the single-cell level. For this purpose, we selected TEM-1 with the least catalytic activity against cefotaxime, followed by its two variants, R164S and G238S, exhibiting moderate and significant catalytic activity, respectively. The results showed that increasing the catalytic activity causes an increase in the population’s mean level of antibiotic resistance. While the type of β-lactamase influences the susceptibility distribution of the strains, this effect is independent of the catalytic activity of the strains. Besides, the genetic characteristics of the strains receiving the β-lactam resistance gene is an important factor that plays a role in the distribution of susceptibility.

## 1. Introduction

According to World Health Organization predictions, antimicrobial-resistant infections could cause 10 million deaths each year by 2050 [1]. The most prevalent bacterial infections that were once treatable have become especially dangerous due to antibiotic resistance diminishing the efficacy of common antibiotics.

Antibiotic resistance occurs when even a few bacteria survive the antibiotic treatment. Phenotypic heterogeneity refers to functional diversity within genetically identical cells, which can enable the division of labour and cooperative behaviours or function as a bet-hedging strategy to enhance the overall survival of the population [2]. While genetic mechanisms of drug resistance are well studied [3], little is known about the basis of the heterogeneous phenotypic response of bacteria to antibiotics.

Heteroresistance is a type of phenotypic heterogeneity wherein seemingly susceptible isogenic bacterial populations contain resistant sub-populations to a particular drug [4]. It is widely prevalent in pathogenic bacteria [5] and can moderate the efficacy of antibacterial infection treatment [6].

Heteroresistance within a bacterial population is detected using the standard reference method, population analysis profiling (PAP), which evaluates the size of the population surviving at concentrations at least eight times higher than the minimum inhibitory concentration (MIC) of the initial population [7–12]. However, this method does not allow for detecting and observing the heteroresistance at the single-cell level within the bacterial population. Since the exact mechanisms of heteroresistance and its role in adaptation to stresses induced by antibiotics are not yet fully understood at the molecular and single-cell level [13], it is crucial to establish a method that enables studies of heteroresistance at the single-cell level. Previously, we have developed a droplet-based approach to determine antibiotic resistance profiles in isogenic bacterial populations originating from a single colony, called clonal heteroresistance [14].

One of the most common types of antibiotic resistance, causing significant economic and societal health-related damages annually, is resistance to β-lactam antibiotics, conveyed by various β-lactamases [15,16]. Based on conserved and distinguishing amino acid motifs β-lactamases are grouped into four classes [17], each affecting a specific range of β-lactams [15,16]. Class A β-lactamases are among the most prevalent classes of β-lactamases and are widely distributed in Gram-negative bacteria, including key ESKAPEE pathogens such as *Klebsiella pneumoniae*, *Acinetobacter baumannii*, *Pseudomonas aeruginosa* and *Enterobacter* species, as well as *Escherichia coli*, which is occasionally included in this category [18]. TEM β-lactamases, which belong to class A β-lactamases, can efficiently hydrolyse penicillins and early-generation cephalosporins, although they have less catalytic activity to oxyimino-cephalosporins such as cefotaxime [18]. TEM β-lactamases are an outstanding example of how β-lactam antibiotics selection pressure led to various mutations in β-lactamases and, subsequently, the emergence of enzymes with more efficient catalytic activity [19]. For instance, so far, more than 200 TEM-1 variants hydrolysing oxyimino-cephalosporins with remarkable efficiency, known as TEM extended spectrum β-lactamase (ESBL), have been identified [18]. In some cases, the substitution of one amino acid in positions such as 164 and 238 can provide the required space for better binding of cefotaxime to the active site of TEM-1, ultimately leading to the improvement of the hydrolysis process [20,21].

In this study, we investigated the impact of the β-lactamase catalytic activity (TEM-1 wild type and its two variants, R164S and G238S), as well as the genetic characteristic of host strains on the distribution of clonal heteroresistance at the single-cell level. To quantify the clonal heteroresistance at the single-cell level, understood here as the distribution of susceptibility at the single-cell level within the bacterial population, we employed a strategy combining droplet microfluidics with fluorescence microscopy. We utilized microfluidic channels to encapsulate single bacterial cells in nanolitre droplets in a high-throughput manner by dispersing aqueous bacterial cultures into an oil phase. Contrary to bacterial cultures carried out at a population level, where crosstalk or mutual support occurs among individuals, bacterial cells originating from a single colony, each encapsulated individually in nanolitre droplets, may independently form their unique populations due to the disruption of bacterial interactions [22–27]. This unique methodological feature allows us to observe the response of individual bacterial cells to the antibiotics based on their characteristics, and, thus, study clonal heteroresistance.

Our findings imply that the distribution of susceptibility at the single-cell level within bacterial population is primarily dependent on the genetic background of the host strain, with additional contributions from the type of TEM family enzyme. Remarkably, the catalytic activity of the strains did not significantly affect the susceptibility of distribution, which suggests the importance of other intrinsic molecular mechanisms. The results provide valuable insights into the complex interactions between bacterial strains and β-lactamase enzymes in the field of antibiotic resistance. The outcomes of our study contribute to a better understanding of the resistance mechanisms, implicating it in the development of more efficient drugs or innovative therapies.

## 2. Results

### 2.1. Selection of ESBL variants and preparation of DNA constructs

**Figure 1.**
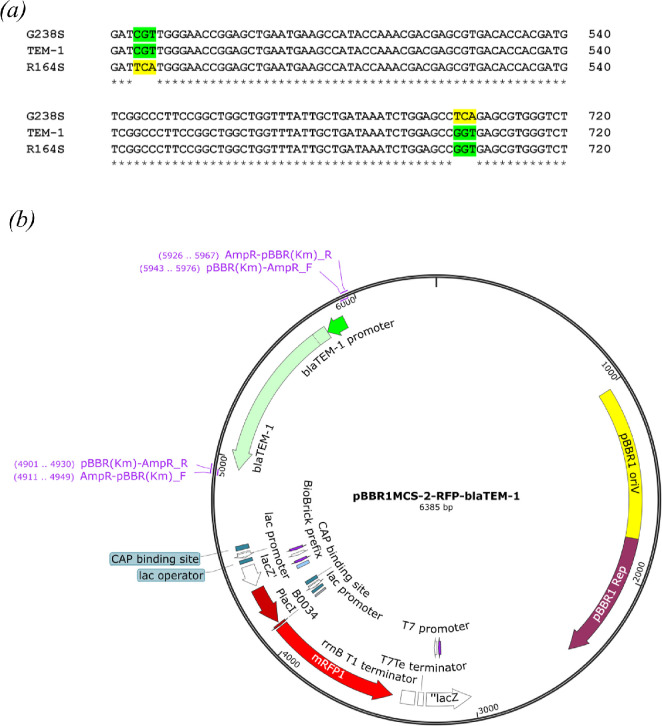
TEM family variants and DNA construct. (*a*) Partial nucleotide sequence of different variants of members of the TEM family used in this study. Highlighted nucleotides are nucleotides that are different than that of TEM-1. Substituted nucleotides are highlighted in yellow. (*b*) The map of vector containing the inserted gene of interest, which in this case is the bla_TEM-1_ gene and its variants, R164S and G238S, under the expression of the bla_TEM-1_ promoter.

The hydrolysis of an antibiotic can be influenced by the type of signal peptide, the strength of the promoter and the size of the resistance gene [28–30]. To minimize the impact of these factors, consequently enabling the examination of only the effect of catalytic activity of β-lactamases on the single-cell distribution of susceptibility within the bacterial population, we carefully selected the enzymes for our study. TEM-1 was used as a wild-type enzyme along with two clinically relevant variants of R164S and G238S (figure 1*a*) [21]. To generate R164S and G238S variants, respectively, arginine at the position of 164 and glycine at the position of 238 were substituted with serine. R164S is able to hydrolyse cefotaxime with five times higher catalytic activity (kcat/KM) than TEM-1, while the efficiency of G238S is reported to be about 80 times higher than TEM-1 [18].

Based on previous studies on bacterial population heterogeneity [31,32], and to reflect clinically observed conditions in our research, the low copy number plasmid pBBR1MCS-2 was used to introduce the resistance genes (figure 1*b*). Utilizing a low copy number plasmid eliminates the possibility of high fluctuation in plasmid numbers [33], and prevents the excessive overexpression of the TEM-1 resistance gene (or its variants) encoded on the plasmid. The plasmid pBBR1MCS-2 carries the red fluorescent protein (mRFP1) gene constitutively expressed along with TEM-1, allowing tracking of the number of viable bacterial cells enclosed in droplets using a fluorescent microscope.

### 2.2. Susceptibility of constructed strains to cefotaxime based on MIC

**Figure 2.**
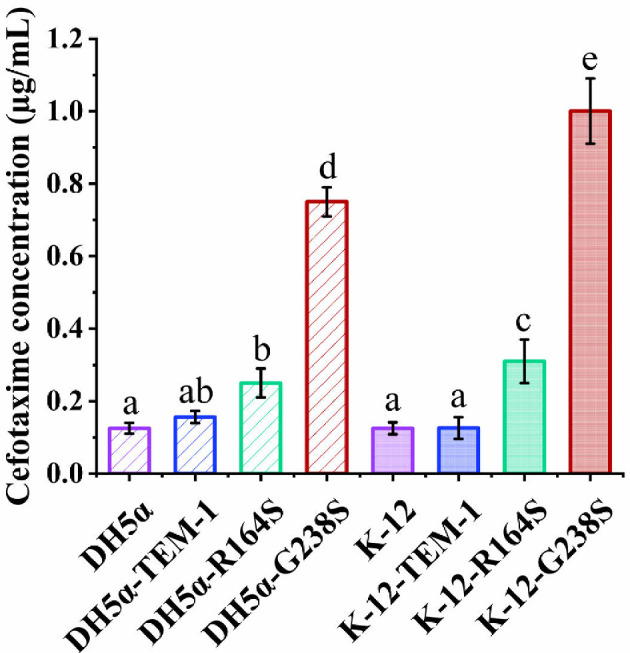
MIC values of cefotaxime against tested strains. MIC values of DH5α- and K-12-strains as a naive strain along with the cells carrying a variant of TEM family member. Bar charts illustrating the MIC mean values for different strains along with associated significance letters denoting pairwise differences (one-way ANOVA, Tukey’s HSD test, *p*‐value < 0.05). Means denoted by a different letter indicate significant differences. The data represent the combined results of three independent experiments.

To examine the effect of the genomic characteristics of the strain on the distribution of the susceptibility of the bacterial population, two different susceptible *E. coli* strains, DH5α and K-12 were used. Analysis of the results from the MIC determination experiments revealed that wild-type *E. coli* DH5α and K-12 strains (containing the pBBR1MCS-2 plasmid without TEM-1 or its variants genes) had similar MIC values, as their growth was inhibited at 0.125 μg ml^−1^ concentration of cefotaxime (figure 2). The MIC value of cefotaxime did not increase by transforming wild type *E. coli* K-12 strain with the pBBR1MCS-2 vector encoding *bla*_TEM-1_ gene, while *E. coli* DH5α cells expressing TEM-1 had a higher MIC value than the wild-type strain. As expected, both *E. coli* DH5α and K-12 cells expressing TEM-1 variants R164S or G238S exhibited increased resistance to cefotaxime compared with the wild-type strains (figure 2). Moreover, the TEM-1 G238S variant conferred higher resistance to cefotaxime in both *E. coli* DH5α and K-12 than the TEM-1 R164S variant. When comparing two strains expressing R164S and G238S, the *E. coli* K-12 strain demonstrated a higher cefotaxime MIC value than *E. coli* DH5α. The difference between MIC values of cefotaxime against *E. coli* strains K-12-TEM-1 and K-12-R164S was about 1.5-fold higher than that of DH5α.

### 2.3. Plasmid copy number among constructed strains

**Figure 3.**
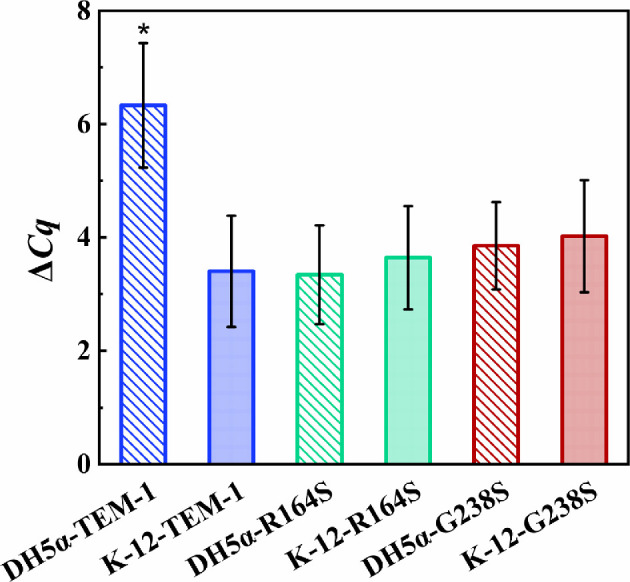
Relative quantification of plasmid copy number in each strain. The asterisk shows the significance at *p* < 0.05 (one-way ANOVA, Tukey HSD test). The results derived from three experiments.

To investigate the extent of the influence of plasmid copy number on the bacterial response to cefotaxime, we conducted relative quantification of the amount of plasmid in *E. coli* DH5α and K-12 strains containing the pBBR1MCS-2 vector with- or without the *bla*_TEM-1_ gene and R164S or G238S variants. The difference in copy number between the β-lactamase gene carried on the plasmid and the *gapA* gene in the chromosome for each strain was determined and compared with those of other strains (figure 3). Analysis of the results showed that the plasmid copy number was significantly increased only in *E. coli* DH5α-TEM-1 strain.

### 2.4. Definition of terms for the analysis of the susceptibility of individual cells

**Figure 4.**
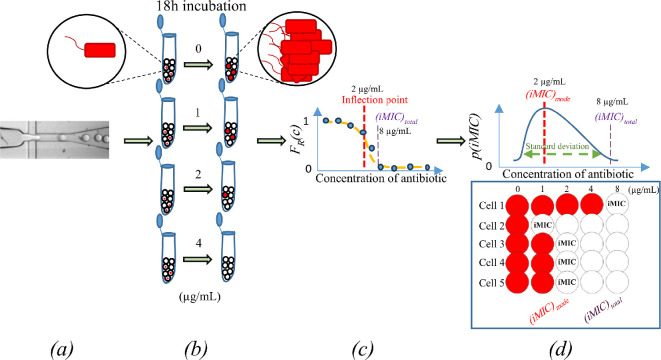
Steps of our study and its terms for analysing the susceptibility of single cells. (*a*) Individual cells, along with culture medium and different concentrations of antibiotic, are encapsulated in oil in water nanolitre-droplets using flow focusing microfluidic method. (*b*) The nl-droplets are collected in 0.2 ml PCR tubes and incubated for 18 h at 37°C. (*c*) The viability profile of individual cells is measured and calculated as explained in §6 of this study. In the diagram, *F*_*R*_*(c)* stands for the fraction of cells that proliferate. (*d*) The inflection points of the graph fitted with the Gompertz function are considered as *(iMIC)*_*mode*_, since they form the top of the curve when the probability of distribution is derived from the viability results. Therefore *p(iMIC)* is the negative value of fraction (*c*) plotted in step (*c*). The end of the curve’s right tail is the concentration of antibiotic inhibiting the growth of all the individual cells, termed *(iMIC)*_*all*_. *(iMIC)*_*total*_ is the minimum antibiotic concentration inhibiting the growth ≥95% of all individually cultured bacteria. The depicted standard deviation of *p(iMIC)* is the dispersion of iMIC values on the *p(iMIC)* curve expected around its mean.

The conventional MIC is defined as the lowest amount of antibiotic, at which the visible growth of bacteria is inhibited. Artemova *et al.* [34] state that the single-cell MIC (scMIC) is the minimal concentration of antibiotic that inhibits bacterial growth in the limit of small densities using dilution of inoculum. Here, we calculate the MIC of an antibiotic needed to inhibit the growth of an individual bacterium. To avoid misinterpretation, we name it individual MIC (iMIC). To determine the iMIC, we encapsulated a single bacterium per 1 nl droplet, by feeding the initial bacterial culture into a microfluidic flow-focusing device (figure 4*a*,*b*). To minimize the probability of encapsulating two or more cells in a single droplet, the initial bacterial culture was diluted to the concentration approximately 1 × 10^5^ CFU ml^−1^, which, based on Poisson distribution, results in around 9% of positive droplets derived from the single bacterium. Following a Poisson distribution, 90% of the total number of droplets, denoted as *N(c)*, consisted of bacteria-free droplets, rendering the growth and, therefore, the relative fluorescence intensity undetectable.

The calculated fractions of individual cells that proliferate into colonies (positive droplets) as a function of antibiotic concentration, *F*_*R*_*(c)*, at each tested antibiotic concentration were fitted into the Gompertz function [14,35,36], as explicitly explained in §6 (figure 4*c*).

We further retrieve the probability density distribution *p(iMIC)*, which of a given cell having a particular value of iMIC, as the negative of the first derivative of *F*_*R*_*(c)* (figure 4*d*)*.* It is important to note that our calculation of *p(iMIC)* from *FR(c)* represents an interpretation step rather than a direct measurement. The derivation of *FR(c)* helps infer the population heterogeneity, providing insight into how bacterial susceptibility is distributed at the single-cell level. The *p(iMIC)* enables the determination of a population’s spread by calculating the standard deviation. The maximal point of the curve refers to the inflection point on the Gompertz function fitted to the experimental data, defining the *(iMIC)*_*mode*_ value. Hence, *(iMIC)*_*mode*_ is the most probable concentration of an antibiotic at which the major number of individually cultured bacteria exhibit their iMIC value. Therefore, *(iMIC)*_*mode*_ is the most probable antibiotic concentration that most of cells show susceptibility to the antibiotic treatment. The minimum antibiotic concentration inhibiting the growth ≥95% of all individually cultured bacteria is termed the total iMIC (*(iMIC)*_*total*_).

### 2.5. Droplet analysis to determine iMIC values

**Figure 5.**
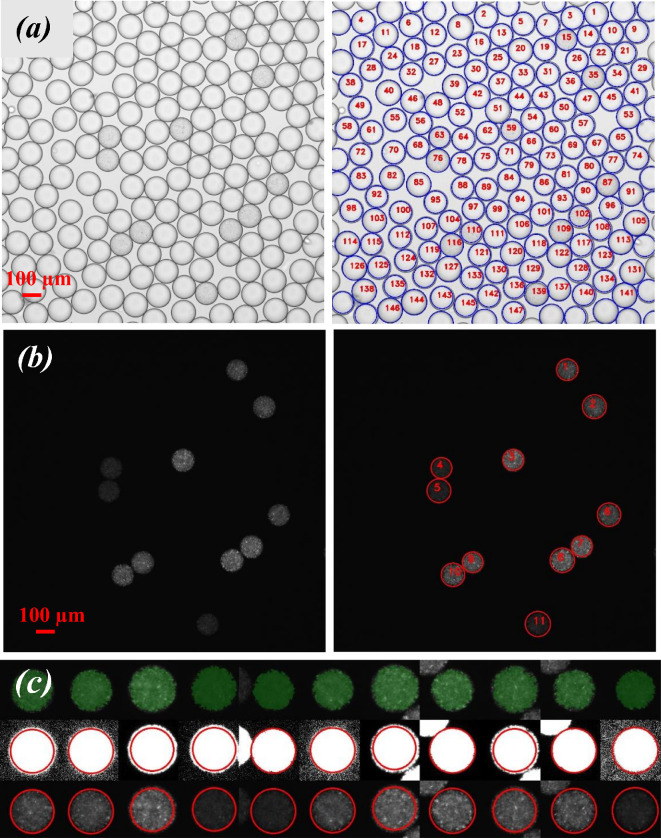
Analysis of droplets. On average, 10 000 droplets were imaged for each cefotaxime concentration, and all experiments were performed in triplicate. (*a*) Droplets showing by bright-field images. (*b*) Fluorescent imaging of positive droplets. (*c*) Detecting the positive droplets and measuring their intensity using image analysis software.

The total number of droplets was directly counted from images obtained from bright-field microscopy and software analysis (figure 5*a*). The number of positive droplets containing grown bacteria, was identified by fluorescent microscopy due to the fluorescence emitted during the expression of mRFP1 (figure 5*b*). The mean fluorescence intensities from droplets were measured by merging the images taken by bright-field and fluorescent microscopy (figure 5*c*). Therefore, the location of the positive droplets containing fluorescent cells was identified with the help of bright-field images using the software. Minor displacements caused by switching the imaging from bright-field to fluorescent microscopy could be identified by the software which properly aligned both images.

### 2.6. Distribution of cefotaxime iMIC in *E. coli* K-12 and DH5α and strains producing the TEM-1, G238S and R164S variants

Since our method to detect positive droplets relies on the fluorescence emitted during the expression of mRFP1 present in pBBR1MCS-2, we were not able to analyse naive strains with no pBBR1MCS-2 for single-cell studies. Further, the use of pBBR1MCS-2 backbone with no resistance gene was avoided, as it could increase the risk of plasmid loss during bacterial cell division [37].

Considering the reasonable relationship between MIC and the catalytic activity of studied β-lactamases, we continued our studies by the comparing *E. coli* K-12 and DH5α strains containing TEM-1, G238S and R164S resistance genes inside the pBBR1MCS-2.

**Figure 6.**
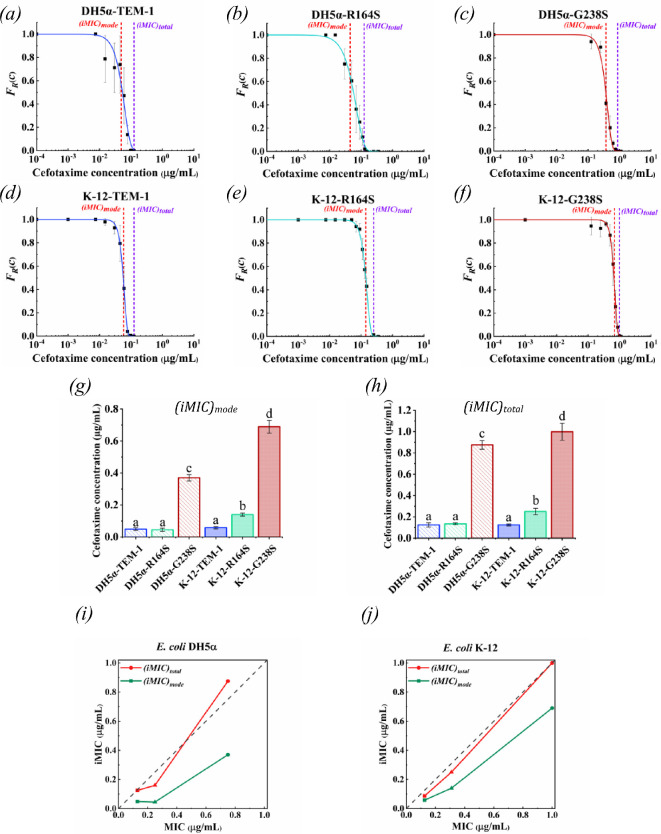
Susceptibility of strains based on individual cells exposed to a specific concentration of cefotaxime inside the nl-droplets. Fraction of resistant bacterial individual cells within the population of tested strains DH5α-TEM-1 (*a*), K-12-TEM-1 (*b*), DH5α-R164S (*c*), K-12-R164S (*d*), DH5α-G238S (*e*) and K-12-G238S (*f*) as a function of an antibiotic concentration. Comparison of *(iMIC)*_*mode*_ (*g*) and *(iMIC)*_*total*_ (*h*) of cefotaxime against tested strains (statistically analysed by one-way ANOVA, Tukey’s HSD test, *p*‐value < 0.05). Consecutive letters denote groups with significant differences in means. The data represent the combined results of three independent experiments. MIC versus iMIC plot for *E. coli* DH5α (*i*) and *E. coli* K-12 (*j*). Red lines demonstrate MIC versus *(iMIC)*_*total*_ and green lines demonstrate MIC versus *(iMIC)*_*mode*_. Squares are strains carrying TEM-1. Triangles are strains carrying R164S. Circles are strains carrying G238S.

Based on the probability density distribution of iMIC (*p(iMIC)*) for each tested strain in the range of cefotaxime concentrations, the *(iMIC)*_*mode*_ of cefotaxime corresponds to the inflection point which refers to the antibiotic concentration that inhibited the growth of the largest number of bacteria (electronic supplementary material, figure S1). In consistency with MIC, iMIC was also related to the catalytic activity of β-lactamase enzymes (figure 6). The value of *(iMIC)*_*mode*_ in *E. coli* K-12 strains expressing R164S and G238S compared with their counterparts expressing TEM-1 increased from 0.058 μg ml^−1^ to 0.14 μg ml^−1^ and 0.69 μg ml^−1^, respectively. In the case of *E. coli* DH5α strains, the value of *(iMIC)*_*mode*_ in G238S expressing strain compared with TEM-1 expressing strain increased from 0.049 μg ml^−1^ to 0.37 μg ml^−1^, while *(iMIC)*_*mode*_ insignificantly decreased to 0.045 μg ml^−1^ in *E. coli* DH5α-R164S (figure 6*g*; table 1). The *(iMIC)*_*total*_ values of cefotaxime followed the same trend as *(iMIC)*_*mode*_ values (figure 6*h*; table 1): the concentrations of cefotaxime that completely inhibited the growth of *E. coli* K-12 strains expressing R164S and G238S were significantly higher than *(iMIC)*_*mode*_ values in corresponding *E. coli* DH5α strains, while the *(iMIC)*_*mode*_ values of cefotaxime in *E. coli* strains expressing TEM-1 were at comparable levels.

**Table 1.** Measures to describe properties of the probability density distribution of iMIC for DH5α- and K-12-strains carrying TEM-1 or its variants R164S and G238S. *γ*, skewness; *K*, kurtosis.

strain		*(iMIC)*_*mode*_	*(iMIC)*_*total*_	*γ*	*K*
DH5α-	TEM-1	0.049	0.125	0.46 (±6.7e−09)	2.97 (±9.5e−09)
	R164S	0.045	0.16	0.78 (±5.4e−09)	3.57 (±3.6e−10)
	G238S	0.37	0.875	0.14 (±2.6e−09)	2.72 (±1.3e−08)
K-12-	TEM-1	0.058	0.087	−0.189 (±4.9e−09)	2.82 (±9.1e−11)
	R164S	0.14	0.25	−0.0412 (±1.5e−09)	2.73 (±1.3e−10)
	G238S	0.69	1	−0.291 (±3.7e−11)	2.92 (±1.4e−11)

In all strains, the *(iMIC)*_*total*_ was proportional to MIC (figure 6*i*,*j*). Apart from the K-12 strain with G238S and the DH5α strain with TEM-1, the MIC and *(iMIC)*_*total*_ values for the remaining strains exhibited minor variations. Also, except for DH5α-R164S, *(iMIC)*_*mode*_ was proportional to MIC in all strains (figure 6*i*,*j*).

### 2.7. Single-cell susceptibility distribution of strains

In all strains, the skewness (*γ*) of the probability density distribution of iMIC (p(iMIC)) can be used to determine the asymmetry of the curve (figure 4*d*). This provides insights into whether the susceptibility distribution of the individual cells in response to an antibiotic is more pronounced at antibiotic concentrations located at the left or right side of the *(iMIC)*_*mode*_ value. Negative skewness values indicate that the distribution is skewed to the left, suggesting the bacterial response is more heterogeneous at concentrations below the *(iMIC)*_*mode*_ value. In other words, in a negatively skewed unimodal distribution, the left side, relative to the *(iMIC)*_*mode*_, covers a broader range of antibiotic concentrations compared with the right side of the distribution. From this, we conclude that the bacterial response to antibiotics is more varied on the left side of the distribution than on the right, in relation to the *(iMIC)*_*mode*_. The calculated skewness for the *p(iMIC)* distribution in K-12 strains was negative (table 1), indicating a longer left tail relative to the right tail (electronic supplementary material, figure S1). On the other hand, positive skewness was observed for the *p(iMIC)* distribution in *E. coli* DH5α strains (table 1), indicating a wider range of antibiotic concentrations that inhibit the proliferation of individual bacteria into colonies on the right side of the distribution than on the left, in relation to the *(iMIC)*_*mode*_. This suggests that a more heterogeneous response is observed at antibiotic concentrations higher than the *(iMIC)*_*mode*_ value. Additionally, positive skewness for the *p(iMIC)* distribution suggests the presence of a small subpopulation of bacteria that is more resistant to the antibiotic than most cells. In other words, as the concentration of cefotaxime increases, the clonal heteroresistance of the bacterial population also increases. The skewness analysis of *p(iMIC)* revealed that the increment of the clonal heteroresistance below or above *(iMIC)*_*mode*_ value is not influenced by the increase in hydrolytic activity of the TEM-1 enzyme but rather by the type of strain (table 1).

**Figure 7.**
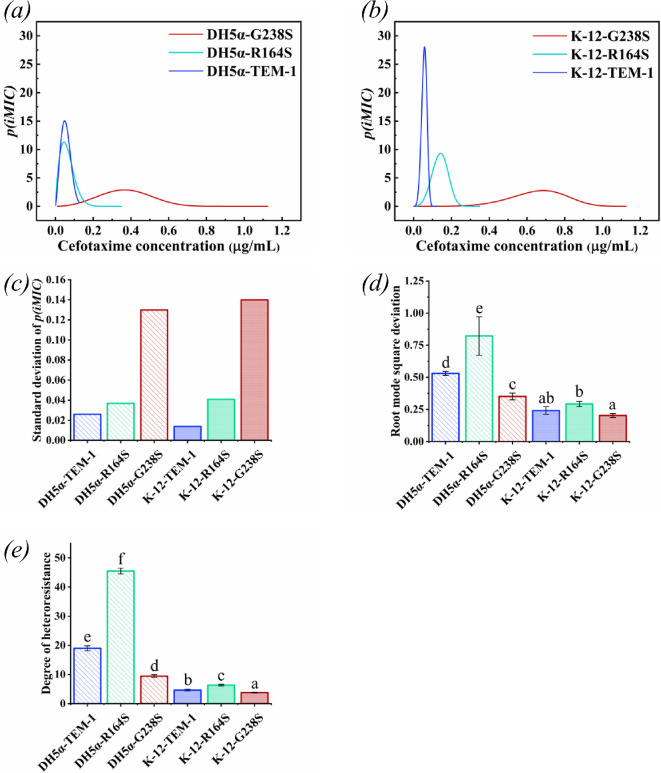
Probability of distribution derived from the iMIC profile of different strains. Distribution probability of (*a*) DH5α and (*b*) K-12 carrying different variants of TEM family. (*c*) The plot represents the standard deviation of *p(iMIC)* for different strains. (*d*) The plot represents root mode square deviation: $=\frac{\sigma }{Mo}$, where *σ* stands for standard deviation of *p(iMIC)* and *Mo* stands for *(iMIC)*_*mode*_. (*e*) The plot illustrates the propensity of each strain to form a heteroresistant population. The degree of heteroresistance is calculated as the ratio of minimum concentration of cefotaxime that inhibits the growth of all individually encapsulated cells by the minimum concentration at which growth inhibition started as indicated by Gompertz function. The data represent the combined results of three independent experiments.

Both, the DH5α as well as K-12, *E. coli* strains expressing the G238S variant had the highest *p(iMIC)* standard deviation compared with those expressing R164S and TEM-1 (figure 7*a–c*). Moreover, the strains carrying the TEM-1 gene had the lowest *p(iMIC)* standard deviation. The K-12-G238S strain with 0.14 had a slightly higher standard deviation of *p(iMIC)* value than the DH5α-G238S with a value of 0.13 (figure 7*c*). The pattern was the same for K-12-R164S with a value of 0.041 and DH5-R164S with a value of 0.037. On the contrary, the DH5α-TEM-1 strain had a standard deviation of *p(iMIC)* value equal to 0.026, which was approximately twice that of the K-12-TEM-1 strain, with a standard deviation of *p(iMIC)* equal to 0.014 (figure 7*c*).

Since each tested strain showed different resistance to cefotaxime, we used the standardized measure of the dispersion of a probability distribution based on the *(iMIC)*_*mode*_ (here is called root mode square deviation) for a more accurate comparison (figure 7*d*). The highest and lowest variations belonged to *E. coli* DH5α-R164S and K12-G238S with 0.82 and 0.2 values of root mode square deviation, respectively. We observed higher root mode square deviation values for the *E. coli* DH5α strain than the K-12 strain. Additionally, both the tested *E. coli* strains containing the R164S allele had more variation than those that carried other alleles. In the next rank were the strains expressing TEM-1, and lastly, the strains carrying G238S were considered as the strains with the lowest variation (figure 7*d*). The results of the root mode square deviation analysis for bacterial population derived from a single cell resemble the results of the analysis of the degree of heteroresistance calculated by the classical definition of population heteroresistance. To determine the degree of heteroresistance, the minimum concentration of cefotaxime that inhibits the growth of all the cells *(iMIC)*_*all*_) was divided by the minimum concentration at which growth inhibition started. This starting point was defined as the concentration where the fraction of proliferating cells exhibited a statistically significant decline from its maximum value, as indicated by the Gompertz function in figure 6*a*–f. Therefore, only the strains of *E. coli* DH5α expressing the TEM-1 and its variants, R164S and G238S, met the classical definition of a heteroresistant population, with 19-, 45.5- and 9.46-fold higher *(iMIC)*_*all*_ than the highest non-inhibitory concentration, respectively (figure 7*e*).

### 2.8. Mean intensity of each droplet

**Figure 8.**
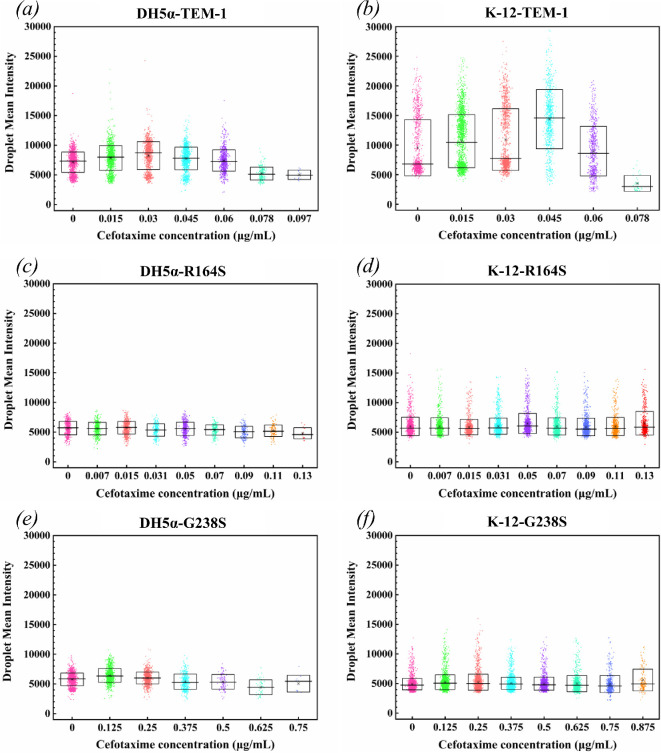
Fluorescence of each droplet, containing the bacterial population of the tested bacterial strain at a given cefotaxime concentration. Droplet mean intensity for *E. coli* strains: (*a*) DH5α-TEM-1, (*b*) K-12-TEM-1, (*c*) DH5α-R164S, (*d*) K-12-R164S, (*e*) DH5α-G238S and (*f*) K-12-G238S. The data represent the combined results of three independent experiments.

To explore the potential relationship between the distribution of susceptibility within the population and heterogeneity of growth, we measured and analysed the mean intensity of each droplet at given concentrations of cefotaxime (figure 8). Based on the results of the plasmid copy number (figure 3), fluorescence changes observed in *E. coli* DH5α-TEM-1 (figure 8*a*) may be primarily influenced by the plasmid copy number. On the other hand, changes in the mean intensity of other strains can be related to the bacterial population inside the droplets.

Strains carrying TEM-1 exhibited high population diversity (figure 8*a*,*b*), although population diversity remained low at high concentrations. The highest population diversity was observed in *E. coli* K-12-TEM-1 (figure 8*b*), mainly between 5000 and 15 000 of the mean intensity. By contrast, the *E. coli* strains expressing R164S and G238S TEM-1 variants (figure 8*c–f*) displayed lower population diversity, with a mean intensity between 5000 and 7000.

## 3. Discussion

Although bacteria often exist in their living environment as a consortium, it is still possible that they be found individually in the environment under certain conditions such as aquatic and soil environments contaminated with antibiotics, as well as using incorrect doses of antibiotics or incomplete antibiotic chemotherapy of infections [38–40]. This is relevant because even a bacterium that has survived antibiotic treatment retains the ability to establish an infection [41]. Therefore, knowing the potential of a bacterium in relation to how it responds to the stresses in the environment at the single-cell level is of great fundamental interest and importance in order to control diseases or prevent the establishment of newer and more resistant mutations.

Moreover, most studies on β-lactamases focus on the enzymatic characteristics or their clinical relevance. Studies that investigate the effect of β-lactamases on the bacterial population are very rare. Conducting the *in vivo* studies can be different than *in vitro* conditions because each cell or strain, according to its characteristics, can respond differently to a certain stimulus or antimicrobial agent, regardless of carrying a specific resistance gene. These responses include but are not limited to the upregulation of stress response genes, efflux pumps, genes responsible for biofilm formation, or the different virulence factors [42–48]. This can be the cause of discrepancies related to a common subject in studies conducted on different strains [49].

In this study, we selected three variants of β-lactamase with different catalytic activity, but with structural characteristics that are mostly close to each other and similar expression elements. Also, we conducted our studies in two *E. coli* strains with considerable differences in their own molecular features to reach a comprehensive conclusion related to the purpose of our study [50]. Thereafter, we mainly focused on the single-cell level of MIC and the susceptibility distribution within the bacterial population, as considered in our previous studies [14].

In conventional methods, where the measurement of bacterial sensitivity is conducted based on the inoculation of a primary bacterial population, it is difficult to find out that the decrease in optical density is caused by the decrease in growth rate or bacterial death. However, in our study, the method used is independent of the growth rate of each bacterium or the number of bacteria present in each droplet at the given time and follows an all-or-nothing system. Therefore, the results can be largely attributed to either survival or lack of survival of the initial population. Fluorescence microscopy-based single-cell analysis, both on agarose and within microfluidic systems, enables rapid assessment of antibiotic response by tracking cellular landmark changes, offering high-resolution insights into bacterial adaptation and heterogeneity [25,27]. Unlike the mother machine [26], nano-droplets provide an isolated, controlled microenvironment for each individual cell. Our setup enables the monitoring of each cell’s independent growth dynamics and improves precision in analysing single-cell responses. By contrast to the method presented by Zagajewski *et al.* [27], our nano-droplet approach using microfluidics is grounded in the concept of MIC determination, moreover we introduce an even more precise measurement called the iMIC value.

Although this method cannot distinguish resistant populations from persistent populations that are metabolically active at a very low level [51], it meets our need in relation to showing the iMIC and the heterogeneity of resistance of the primary population in response to different concentrations of cefotaxime. Additionally, our comparative analysis of different strains enabled us to reach conclusions independently of control results, as the relative comparison alone was sufficient for our conclusions. Based on the aim of this study, the bacterial population in the interval between the lowest concentration of antibiotic, at which growth inhibition begins, and the lowest concentration, at which growth is completely inhibited, is of great importance. This remaining population is the number of bacteria from the primary population that shows a higher resistance over the rest of the population at a certain concentration of antibiotic.

Based on the results, both naive *E. coli* strains of DH5α and K-12 show similar susceptibility to cefotaxime (figure 2), but the presence of similar β-lactamase variants in each of the strains led to noticeable differences in the level of resistance. All strains, except *E. coli* DH5α-TEM-1, had an increase in the resistance which was proportional to the catalytic activity of the received variant of the TEM family. Considering the fact that in *E. coli* DH5α-TEM-1 the plasmid copy number is about double that of other strains (figure 3), it can be concluded that the increase in the hydrolysis potential of cefotaxime increases the value of both MIC and iMIC (figures 2 and 6; electronic supplementary material, figure S1). In such a way, the increase in plasmid copy number can increase the production of TEM-1 which consequently increases the cefotaxime hydrolysis by *E. coli* DH5α strain [29,52]. Therefore, it shows an *(iMIC)*_*mode*_ value close to R164S, with statistically insignificantly higher catalytic activity, due to this compensation. The main reason that *E. coli* DH5α-TEM-1 has a higher plasmid copy number compared with other strains remains unclear, but this feature can be attributed to its different segregational plasmid instability under the conditions of our study [53].

Additionally, the unique physiological characteristics of *E. coli* DH5α- and K-12-naive strains can be an explanation for the differences in the resistance acquired through carrying R164S and G238S, when compared with each other. One notable difference between the two strains is the inactivation of RecA in DH5α [54], which plays a role in the repair of DNA damage [55]. Other key differences include mutation in RelA, which is involved in the cellular stress response [56]. These genetic differences may contribute to the higher resistance observed in the K-12-G238S and K-12-R164S strains compared with their DH5α counterparts. Since the initial concentration of cefotaxime used to test the strains carrying R164S and G238S mutations was higher than that used for cells carrying TEM-1, this elevated concentration of cefotaxime may induce increased stress, potentially leading to DNA damage [57,58]. The activity of RecA, along with the role of mutated genes such as *relA*, may enable K-12 strains to better tolerate these higher cefotaxime concentrations compared with DH5α strains.

Moreover, the analysis of our results showed that there is a significant relationship between the type of strains and the heterogeneity of their resistance (figure 7). For example, the DH5α-G238S strain, which had the lowest distribution of susceptibility among all DH5α strains with a root mode square deviation of 0.35, had a higher distribution of susceptibility than K-12-R164S with a root mode square deviation of 0.29, which had the highest heterogeneity of resistance among all K-12 strains.

Further, each TEM family enzyme affects the level of clonal heteroresistance regardless of its catalytic activity. Based on our results, the R164S allele, which exhibits moderate catalytic activity against cefotaxime, resulted in a broader distribution of susceptibility compared with TEM-1, which has lower catalytic activity, and G238S, which has higher catalytic activity, in both DH5α and K-12 (figure 7). The analysis of the results showed that the combination of DH5α strain and R164S variant can contribute to the emergence of a population with the highest heteroresistance compared with the other strains studied here. This suggests that strain-specific-genetic characteristics may trigger distinct molecular mechanisms, exhibiting considerable variability both between strains and even among individual cells [41,59,60]. Therefore, we speculate that in such a way, the genetic content of bacterial strains plays a role in increasing the heterogeneity of these molecular mechanisms.

Despite DH5α-TEM-1 having the highest plasmid copy number as determined by PCR, the fluorescence intensity of its droplets was lower than that of the K-12 analogs. Given that both strains exhibit similar MICs, this suggests that the final number of bacteria per droplet (after growth) in K-12 is significantly higher than in DH5α. It has previously been suggested that there is an inverse relationship between bacterial growth heterogeneity and heteroresistance, indicating that an increase in heteroresistance is correlated with a decrease in growth rate [61]. Considering the apparent higher heterogeneity in growth of the K-12 strains compared with DH5α strains (figure 8), there appears to be an inverse relationship between heterogeneity in growth and susceptibility distribution. These results suggest a complex interplay within bacterial populations and underscores the need for deeper exploration into the regulatory mechanisms and environmental factors influencing these traits.

Apart from our main findings, using the method that we developed, at the same time, antibiotic susceptibility of a large number of bacterial cells from a selected population is investigated, and this profile is used for further studies, including the investigation of clonal heteroresistance. This is significant as prior studies suggested that single-cell MIC is more reliable than MIC due to the lack of an inoculum effect [34]. Our results showed that there is a relationship between *(iMIC)*_*total*_ and MIC. Like MIC, *(iMIC)*_*total*_ increases with increasing catalytic activity of the strain (figure 6). However, not all the strains had a fully match MIC and *(iMIC)*_*total*_ values. In addition, *(iMIC)*_*mode*_, which represents the concentration of cefotaxime most likely to affect the largest number of individually encapsulated bacterial cells, may not follow MIC and *(iMIC)*_*total*_ in some cases. For example, the DH5α-TEM-1 exhibits a stronger correlation between MIC and *(iMIC)*_*total*_ than DH5α-R164S. However, it demonstrates a weaker correlation between MIC and *(iMIC)*_*mode*_ than the latter (figure 6*i*). Besides, the skewness of *p(iMIC)* curves may indicate population heterogeneity. Positive skewness observed in DH5α-TEM-1, -R164S and -G238S strains suggests higher heterogeneity above *(iMIC)*_*mode*_, while negative skewness in K-12-TEM-1 and its variants indicates increased heterogeneity below *(iMIC)*_*mode*_. These strain-specific responses suggest a link between heterogeneity and resistance development, however further experiments are required to confirm these findings.

## 4. Conclusion

In this study, we showed that the distribution of susceptibility within the bacterial population can be largely related to the characteristics of the bacterial strains. For example, the presence or absence of specific stress response proteins that can participate in the emergence of a heteroresistance population. The influence of β-lactamase on the distribution of susceptibility is evidently unrelated to its catalytic activity, although each β-lactamase can affect the level of heteroresistance to some extent. It is usually believed that heterogeneity in resistance causes the emergence of more resistant strains [62]. Also, it has been recently reported that the emergence of a more resistant population occurs at antibiotic concentrations lower than MIC [63,64]. Therefore, it seems that these concentrations, in addition to inducing the necessary stress to change the characteristics of the bacteria, can also provide enough opportunity for the adaptation of cells through specific molecular mechanisms. Our results provide a new perspective on the behaviour of bacteria at the single-cell level in these concentrations, which can be of great help in controlling mutations and new resistance mechanisms. Furthermore, the technique we described in this study can be used as a new way to determine bacterial susceptibility to antimicrobial agents. Importantly, this technique can be used for basic studies related to the effect of different factors on the diversity of the bacterial population.

## 5. Limitations and future directions

While this method effectively assesses individual MIC and the heterogeneity of resistance in the studied strains, several potential improvements can enhance its capacity to evaluate the influence of plasmid copy number, fitness cost and growth heterogeneity. One such improvement involves sorting droplets based on their fluorescence intensity and subsequently measuring their plasmid copy number. Additionally, incorporating a second fluorescent marker, such as GFP, into the chromosome could enable differentiation between growth rate effects and plasmid copy number variations, further refining the analysis. Additional studies with a broader range of MIC and iMIC values using more bacterial strains would be beneficial for fully characterizing this relationship and refining our understanding of its underlying mechanisms.

## 6. Material and methods

### 6.1. Strains, constructs and medium

The backbone of a low copy number plasmid, pBBR1MCS-2 containing a gene responsible for expression of mRFP1 as a reporter [65], was used to insert resistance genes. The mrfp1 gene was placed into the Lac promoter sequence, resulting in the complete shutdown of the Lac promoter’s activity. TEM-1 (GenBank accession WBR59013.1) and its promoter at the upstream were amplified using a pair of appropriate primers listed in table 1, and replaced by the kanamycin resistance gene and its promoter in pBBR1MCS-2 using Gibson assembly Master Mix provided by New England BioLabs (MA, USA). Overall, both TEM-1 and mRFP are produced under the control of the TEM1 promoter. To generate G238S and R164S mutations, a set of primers with appropriate overhangs was designed (table 1) for substituting the codons at the relevant positions. The mutations were confirmed by Sanger sequencing.

The DNA constructs encoding TEM-1 or its variants G238S and R164S were transformed into two strains of *E. coli*, DH5α and K-12, which are susceptible to β-lactam antibiotics. The Mueller Hinton (MH) broth (Difco) was used for antimicrobial susceptibility testing.

### 6.2. Determination of plasmid copy number

Five millilitres of MH broth containing a sub-MIC concentration of cefotaxime were inoculated by 5 × 10^5^ CFU ml^−1^ final concentration and then incubated overnight. The sub-MIC values of cefotaxime against DH5α-TEM-1, DH5α-R164S, DH5α-G238S, K-12-TEM-1, K-12-R164S and K-12-G238S were 0.078, 0.125, 0.375, 0.063, 0.155 and 0.5 μg ml^−1^, respectively. Genomic DNA from the strains was isolated using a Monarch Genomic DNA purification kit (NEB, MA, USA). The quantification cycle (*Cq*) values of the TEM-1 gene and its variants, R164S and G238S, encoded on the plasmid were subtracted from the *Cq* value of the *gapA* gene encoded on the chromosome using quantitative real-time PCR [30]. The primers listed in electronic supplementary material, table S1 were used for this purpose.

### 6.3. Determination of minimum inhibitory concentration (MIC)

One colony from each strain was picked up from LB agar containing 50 μg ml^−1^ ampicillin and transferred to 5 ml of MH broth containing 50 μg ml^−1^ ampicillin and incubated overnight at 37°C, 220 rpm. Then, the bacteria were inoculated to the ratio of 1:1000 v/v into a fresh MH broth containing 50 μg ml^−1^ ampicillin and incubated to reach OD600 ~0.1. The standard inoculum density of 5 × 10^5^ CFU ml^−1^ recommended by CLSI and EUCAST [66] was used for a twofold dilution method.

### 6.4. Design and fabrication of microfluidic device

We used two different microfluidic chips, one with a flow-focusing geometry for the generation of droplets and the other with chambers for imaging (electronic supplementary material, figure S2). A CNC machine (MSG4025, Ergwind, Poland) was used to mill the channels for the droplet generation chip onto a polycarbonate (PC) plate (Macroclear, Bayer, Germany). To prepare the polydimethylsiloxane (PDMS) mold, a 10:1 ratio of prepolymer to curing agent (Sylgard 184, Dow Corning, USA) was thoroughly mixed, poured onto the PC chip and cured at 75°C for 2.5 h. The PDMS mold surface was silanized with vapours of tridecafluoro-1,1,2,2-tetrahydrooctyl-1-trichlorosilane (United Chemical Technologies, USA) for 1 h under 10 mBar pressure. The chip and a glass slide were plasma treated (Harrick Plasma, USA) and bonded together. To achieve hydrophobicity, Novec 1720 (3M, USA) was flushed into the microchannels and allowed to evaporate at room temperature. To further enhance adhesion between glass and PDMS, the chips were baked at 75°C for 1 h.

The chip for the droplet imaging was fabricated on a 4-inch Silicon (Si) wafer using standard photolithography. The geometry and design of the photomask was drawn on AutoCAD (2020, Version 1.4). The fabrication process involved spin coating a negative photoresist SU-8 (Kayaku Advanced Materials, USA) at 3000 rpm to achieve the desired thickness of the channels. The spin-coated wafer was baked at 65°C and 95°C according to the manufacturer’s specifications. The Si master mold was ready after subsequent UV exposure and development. PDMS was poured onto the master mold and cured to obtain PDMS chips which were then bonded to a clean glass slide after plasma treatment.

### 6.5. Microfluidics for droplet generation and image acquisition

Novec HFE-7500 fluorocarbon oil (3M, USA) containing 2% PFPE-PEG-PFPE surfactant was used for making 1 nl water in oil droplets as described previously [14,67]. The flow rate of oil and samples of bacteria with formulated concentrations of antibiotics were controlled using NemeSYS pumps (Cetoni GmBH, Germany) fitted with glass syringes (Hamilton, USA) and operated by software QmixElements (Cetoni GmBH, Germany). The generated droplets from each sample were collected separately in 0.2 ml microtubes for incubation at 37°C.

The bacterial encapsulation density per droplet was calculated based on the Poisson statistics [68]. Based on the calculations an initial inoculum size of 1 × 10^5^ CFU ml^−1^ was used to generate 1 nl droplets for cefotaxime susceptibility testing. About 90% of droplets were predicted with no cells, 9% droplets with single cells and 1% with two or more cells.

The droplets after incubation were aspirated in a polytetrafluoroethylene tubing (AdTech) using the same setup as for droplet generation and, pushed into the imaging chamber. The acquisition was done using an inverted microscope (Nikon Eclipse Ti2), equipped with a camera (Andor Zyla sCMOS), colour selective light source (Lumencor Spectra X) and 10×/0.30 objective (Nikon Plan Flour). Each field of view was captured in mCherry channel (Semrock mCherry-C NKBV=-0014 filter) with excitation 555 nm and emission 635 nm followed by bright-field in quick succession. Fluorescence was measured with a 100 ms exposure time and 5% lamp intensity to prevent pixel saturation, ensuring that pixel counts remained below 60 000 for TIFF format images (16-bit depth). Acquired images were then extracted separately for both channels in 16-bit greyscale using NIS-elements AR software (v5.41.00) for further analysis.

### 6.6. Droplet analysis

We developed our own computer program to perform the droplet image analysis. The software was created using the Python programming language and the Scikit-image library [69]. The bright-field image was used to the calculate total number of droplets in a field of view as well as their positions, and the successive fluorescence image of the same field was used to count droplets containing bacteria and relative droplet fluorescent intensities.

### 6.7. Calculations of individual MIC and the distribution of susceptibility at the single-cell level within the bacterial population

We performed the following calculations to calculate iMIC. First, we determined the total number of droplets, *N(c)*, with a specific antibiotic concentration for each droplet library. We assessed the number of positive droplets, *N*_*+*_*(c)*, defined as droplets containing bacterial populations derived from individual bacteria detectable by relative fluorescence intensity measurements.

The fraction of positive droplets is denoted by *f_+_(c) = N_+_(c)/N(c)*. To analyse the response of bacteria to antibiotics, we normalized the number of positive droplets, which exhibited a positive signal after 18 h of cefotaxime treatment, *f*_*+*_*(c)*, by the total number of positive droplets containing bacteria grown in the absence of antibiotic, *f*_*+*_*(0)*, to obtain the fraction of individual cells that proliferate as a function of antibiotic concentration: *F*_*R*_*(c) = f*_*+*_*(c)/f*_*+*_*(0).* We fitted the experimental data for each fraction of individual cells that proliferate, *F*_*R*_*(c)*, at given antibiotic concentration by the Gompertz function [70,71]:


$$\varphi \left(c\right)=exp\left\{-{\left(c/p_{1}\right)}^{p_{2}}\right\}:(F_{R}(c)=\;\varphi \left(c\right))$$

where *c* represents the antibiotic concentration, *p*_*1*_ is a concentration at a maximum slope and *p*_*2*_ is a slope parameter at *c* = *p*_*1*_.

For estimation of the parameter *s* of the Gompertz function, we use the nonlinear least-squares routines (Levenberg–Marquardt) [72,73].

Using the Gompertz function we calculated the inflection point of the curve. Mathematically, this point represents the second derivative of *F*_*R*_*(c)*, which equals zero for *c = c*_*(iMIC)*_. By solving this *d*^*2*^*F*_*R*_*(c)/dc*^*2*^ *= 0,* we obtain ciMICmode=p11-1p21p2. To calculate the probability density of distribution *p(c)*, that the growth of a bacterium will be inhibited at concentration *c = c_(iMIC)_,* we used the expression *p(c) = - dF_R_(c)/dc*, where: pc=p2cp2-1p1p2exp⁡{-(cp1)p2}

For the function *p(c)*, we calculated the average value *µ*, variance *σ* and skewness *γ*, utilizing the gamma function *Г*(*x*): $µ=\int_{0}^{\infty }dccp(c)$, $\sigma^{2}=$$\int_{0}^{\infty }dc{\left(c-µ\right)}^{2}p(c)$ and $\gamma =\frac{\mu_{3}}{\sigma^{3}}$ where $\sigma^{3}=$$\int_{0}^{\infty }dc{\left(c-µ\right)}^{3}p(c)$ and ${µ}_{3}=$$\int_{0}^{\infty }dc{\left(c-µ\right)}^{3}p(c)$:


$$\mu =\left(p_{1}/p_{2}\right)\Gamma \left(1/p_{2}\right);\sigma =\sqrt{\left(2p_{1}^{2}/p_{2}\right)\Gamma \left(2/p_{2}\right)-\mu^{2}};$$

$$\gamma =\frac{\left[\left(3p_{1}^{3}/p_{2}\right)\Gamma \left(3/p_{2}\right)-\mu \left(3\sigma^{2}+\mu^{2}\right)\right]}{\sigma^{3}}.$$

Analysing the experiment results, we determined *µ* (the average *p(c)* values) and *σ* (their scatter around these values). The skewness *γ* offers insights into the characteristics exhibited by the probability distribution curve.

From the probability density of distribution *p(c)*, we defined the mode of iMIC (*(iMIC)*_*mode*_) as the concentration at which *F*_*R*_*(c)* exhibits the maximum slope. This concentration corresponds to the point where the antibiotic inhibits the growth of the highest number of bacteria with the most significant probability.

## Data Availability

Data for this article, including the code for droplet analysis, code used for the raw data calculations and results for raw data fitting into the Gompertz function are available at RepOD [74]. Supplementary material is available online [75].

## References

[1] World Health Organization. 2019 New report calls for urgent action to avert antimicrobial resistance crisis. See https://www.who.int/news/item/29-04-2019-new-report-calls-for-urgent-action-to-avert-antimicrobial-resistance-crisis (accessed April 2019).

[2] Reyes Ruiz LM, Williams CL, Tamayo R. 2020 Enhancing bacterial survival through phenotypic heterogeneity. PLoS Pathog. **16**, e1008439. (10.1371/journal.ppat.1008439) 32437427 PMC7241687

[3] Davies J, Davies D. 2010 Origins and evolution of antibiotic resistance. Microbiol. Mol. Biol. Rev. **74**, 417–433. (10.1128/MMBR.00016-10) 20805405 PMC2937522

[4] El-Halfawy OM, Valvano MA. 2015 Antimicrobial heteroresistance: an emerging field in need of clarity. Clin. Microbiol. Rev. **28**, 191–207. (10.1128/CMR.00058-14) 25567227 PMC4284305

[5] Nicoloff H, Hjort K, Levin BR, Andersson DI. 2019 The high prevalence of antibiotic heteroresistance in pathogenic bacteria is mainly caused by gene amplification. Nat. Microbiol. **4**, 504–514. (10.1038/s41564-018-0342-0) 30742072

[6] Band VI, Weiss DS. 2019 Heteroresistance: A cause of unexplained antibiotic treatment failure? PLoS Pathog. **15**, e1007726. (10.1371/journal.ppat.1007726) 31170271 PMC6553791

[7] Hung KH, Wang MC, Huang AH, Yan JJ, Wu JJ. 2012 Heteroresistance to cephalosporins and penicillins in Acinetobacter baumannii. J. Clin. Microbiol. **50**, 721–726. (10.1128/JCM.05085-11) 22189112 PMC3295183

[8] Ryffel C, Strässle A, Kayser FH, Berger-Bächi BJA. 1994 Mechanisms of heteroresistance in methicillin-resistant Staphylococcus aureus. Antimicrob. Agents Chemother. **38**, 724–728. (10.1128/AAC.38.4.724) 8031036 PMC284532

[9] Kayser FH, Benner EJ, Hoeprich PD. 1970 Acquired and native resistance of Staphylococcus aureus to cephalexin and other beta-lactam antibiotics. Appl. Microbiol. **20**, 1–5. (10.1128/am.20.1.1-5.1970) 5201887 PMC376855

[10] Sutherland R, Rolinson GN. 1964 Characteristics of methicillin-resistant staphylococci. J. Bacteriol. **87**, 887–899. (10.1128/jb.87.4.887-899.1964) 14137628 PMC277108

[11] El-Halfawy OM, Valvano MA. 2015 Antimicrobial heteroresistance: an emerging field in need of clarity. Clin. Microbiol. Rev. **28**, 191–207. (10.1128/CMR.00058-14) 25567227 PMC4284305

[12] El-Halfawy OM, Valvano MA. 2013 Chemical communication of antibiotic resistance by a highly resistant subpopulation of bacterial cells. PLoS ONE **8**, e68874. (10.1371/journal.pone.0068874) 23844246 PMC3700957

[13] Xu Y *et al*. 2020 Mechanisms of heteroresistance and resistance to Imipenem in Pseudomonas aeruginosa. Infect. Drug Resist. **13**, 1419–1428. (10.2147/IDR.S249475) 32523360 PMC7234976

[14] Scheler O *et al*. 2020 Droplet-based digital antibiotic susceptibility screen reveals single-cell clonal heteroresistance in an isogenic bacterial population. Sci. Rep. **10**, 3282. (10.1038/s41598-020-60381-z) 32094499 PMC7039976

[15] Rice LB, Bonomo RA. 2000 beta -Lactamases: which ones are clinically important? Drug Resist. Updat. **3**, 178–189. (10.1054/drup.2000.0144) 11498383

[16] Bush K. 2013 Proliferation and significance of clinically relevant β-lactamases. Ann. N. Y. Acad. Sci. **1277**, 84–90. (10.1111/nyas.12023) 23346859

[17] Bush K, Jacoby GA. 2010 Updated functional classification of beta-lactamases. Antimicrob. Agents Chemother. **54**, 969–976. (10.1128/AAC.01009-09) 19995920 PMC2825993

[18] Palzkill T. 2018 Structural and mechanistic basis for extended-spectrum drug-resistance mutations in altering the specificity of TEM, CTX-M, and KPC β-lactamases. Front. Mol. Biosci. **5**, 16. (10.3389/fmolb.2018.00016) 29527530 PMC5829062

[19] Gniadkowski M. 2008 Evolution of extended-spectrum beta-lactamases by mutation. Clin. Microbiol. Infect. **14 Suppl 1**, 11–32. (10.1111/j.1469-0691.2007.01854.x) 18154525

[20] Baraniak A, Fiett J, Mrówka A, Walory J, Hryniewicz W, Gniadkowski M. 2005 Evolution of TEM-type extended-spectrum beta-lactamases in clinical Enterobacteriaceae strains in Poland. Antimicrob. Agents Chemother. **49**, 1872–1880. (10.1128/AAC.49.5.1872-1880.2005) 15855509 PMC1087658

[21] Salverda MLM, De Visser JAGM, Barlow M. 2010 Natural evolution of TEM-1 β-lactamase: experimental reconstruction and clinical relevance. FEMS Microbiol. Rev. **34**, 1015–1036. (10.1111/j.1574-6976.2010.00222.x) 20412308

[22] Pacocha N, Bogusławski J, Horka M, Makuch K, Liżewski K, Wojtkowski M, Garstecki P. 2021 High-throughput monitoring of bacterial cell density in nanoliter droplets: label-free detection of unmodified gram-positive and gram-negative bacteria. Anal. Chem. **93**, 843–850. (10.1021/acs.analchem.0c03408) 33301291

[23] Postek W, Garstecki P. 2022 Droplet microfluidics for high-throughput analysis of antibiotic susceptibility in bacterial cells and populations. Acc. Chem. Res. **55**, 605–615. (10.1021/acs.accounts.1c00729) 35119826 PMC8892833

[24] Quellec LL, Aristov A, Ramos SG, Amselem G, Bos J, Baharoglu Z, Mazel D, Baroud CNJ. 2023 Measuring single-cell susceptibility to antibiotics within monoclonal bacterial populations. PLoS ONE **19(8)**, e0303630. (10.1371/journal.pone.0303630) PMC11293721 39088440

[25] Chatzimichail S *et al*. 2024 Rapid identification of bacterial isolates using microfluidic adaptive channels and multiplexed fluorescence microscopy. Lab Chip **24**, 4843–4858. (10.1039/d4lc00325j) 39291847 PMC11409657

[26] Baltekin Ö, Boucharin A, Tano E, Andersson DI, Elf J. 2017 Antibiotic susceptibility testing in less than 30 min using direct single-cell imaging. Proc. Natl. Acad. Sci. U.S.A. **114**, 9170–9175. (10.1073/pnas.1708558114) 28790187 PMC5576829

[27] Zagajewski A *et al*. 2023 Deep learning and single-cell phenotyping for rapid antimicrobial susceptibility detection in Escherichia coli. Commun. Biol. **6**, 1164. (10.1038/s42003-023-05524-4) 37964031 PMC10645916

[28] Fournier B, Gravel A, Hooper DC, Roy PH. 1999 Strength and regulation of the different promoters for chromosomal beta-lactamases of Klebsiella oxytoca. Antimicrob. Agents Chemother. **43**, 850–855. (10.1128/AAC.43.4.850) 10103190 PMC89216

[29] Pereira C, Larsson J, Hjort K, Elf J, Andersson DI. 2021 The highly dynamic nature of bacterial heteroresistance impairs its clinical detection. Commun. Biol. **4**, 521. (10.1038/s42003-021-02052-x) 33953333 PMC8099907

[30] Wang X *et al*. 2014 Heteroresistance at the single-cell level: adapting to antibiotic stress through a population-based strategy and growth-controlled interphenotypic coordination. MBio **5**, e00942–00913. (10.1128/mBio.00942-13) 24520060 PMC3950525

[31] Doijad SP *et al*. 2023 Resolving colistin resistance and heteroresistance in Enterobacter species. Nat. Commun. **14**, 140. (10.1038/s41467-022-35717-0) 36627272 PMC9832134

[32] Panta PR, Doerrler WT. 2021 A link between pH homeostasis and colistin resistance in bacteria. Sci. Rep. **11**, 13230. (10.1038/s41598-021-92718-7) 34168215 PMC8225787

[33] de la Hoz AB, Ayora S, Sitkiewicz I, Fernández S, Pankiewicz R, Alonso JC, Ceglowski P. 2000 Plasmid copy-number control and better-than-random segregation genes of pSM19035 share a common regulator. Proc. Natl. Acad. Sci. U.S.A. **97**, 728–733. (10.1073/pnas.97.2.728) 10639147 PMC15398

[34] Artemova T, Gerardin Y, Dudley C, Vega NM, Gore JJM. 2015 Isolated cell behavior drives the evolution of antibiotic resistance. Mol. Syst. Biol. **11**, 822. (10.15252/msb.20145888) 26227664 PMC4547850

[35] Chorianopoulos NG, Lambert RJW, Skandamis PN, Evergetis ET, Haroutounian SA, Nychas GJE. 2006 A newly developed assay to study the minimum inhibitory concentration of Satureja spinosa essential oil. J. Appl. Microbiol. **100**, 778–786. (10.1111/j.1365-2672.2006.02827.x) 16553733

[36] Lambert RJ, Pearson J. 2000 Susceptibility testing: accurate and reproducible minimum inhibitory concentration (MIC) and non-inhibitory concentration (NIC) values. J. Appl. Microbiol. **88**, 784–790. (10.1046/j.1365-2672.2000.01017.x) 10792538

[37] Lopatkin AJ, Meredith HR, Srimani JK, Pfeiffer C, Durrett R, You L. 2017 Persistence and reversal of plasmid-mediated antibiotic resistance. Nat. Commun. **8**, 1689. (10.1038/s41467-017-01532-1) 29162798 PMC5698434

[38] Thiele‐Bruhn S. 2003 Pharmaceutical antibiotic compounds in soils—a review. Z. Pflanzenernähr. Bodenk **166**, 145–167. (10.1002/jpln.200390023)

[39] Pettersson ME, Andersson DI, Roth JR, Berg OG. 2005 The amplification model for adaptive mutation. Genetics **169**, 1105–1115. (10.1534/genetics.104.030338) 15489536 PMC1449099

[40] Andersson DI, Hughes D. 2014 Microbiological effects of sublethal levels of antibiotics. Nat. Rev. Microbiol. **12**, 465–478. (10.1038/nrmicro3270) 24861036

[41] Coates J, Park BR, Le D, Şimşek E, Chaudhry W, Kim M. 2018 Antibiotic-induced population fluctuations and stochastic clearance of bacteria. Elife **7**, e32976. (10.7554/eLife.32976) 29508699 PMC5847335

[42] Shahryari S, Talaee M, Haghbeen K, Adrian L, Vali H, Shahbani Zahiri H, Noghabi KA. 2021 New provisional function of OmpA from Acinetobacter sp. strain SA01 based on environmental challenges. mSystems **6**, e01175–01120. (10.1128/mSystems.01175-20) 33436517 PMC7901484

[43] Poole K. 2012 Bacterial stress responses as determinants of antimicrobial resistance. J. Antimicrob. Chemother. **67**, 2069–2089. (10.1093/jac/dks196) 22618862

[44] Ebbensgaard AE, Løbner-Olesen A, Frimodt-Møller J. 2020 The role of efflux pumps in the transition from low-level to clinical antibiotic resistance. Antibiotics (Basel). **9**, 855. (10.3390/antibiotics9120855) 33266054 PMC7760520

[45] Alcalde-Rico M, Hernando-Amado S, Blanco P, Martínez JL. 2016 Multidrug efflux pumps at the crossroad between antibiotic resistance and bacterial virulence. Front. Microbiol. **7**, 1483. (10.3389/fmicb.2016.01483) 27708632 PMC5030252

[46] Pages JM, Lavigne JP, Leflon-Guibout V, Marcon E, Bert F, Noussair L, Nicolas-Chanoine MH. 2009 Efflux pump, the masked side of beta-lactam resistance in Klebsiella pneumoniae clinical isolates. PLoS ONE **4**, e4817. (10.1371/journal.pone.0004817) 19279676 PMC2652100

[47] Mah TF, O’Toole GA. 2001 Mechanisms of biofilm resistance to antimicrobial agents. Trends Microbiol. **9**, 34–39. (10.1016/s0966-842x(00)01913-2) 11166241

[48] Høiby N, Bjarnsholt T, Givskov M, Molin S, Ciofu O. 2010 Antibiotic resistance of bacterial biofilms. Int. J. Antimicrob. Agents **35**, 322–332. (10.1016/j.ijantimicag.2009.12.011) 20149602

[49] Lucaßen K, Gerson S, Xanthopoulou K, Wille J, Wille T, Seifert H, Higgins PG. 2021 Comparison of the Acinetobacter baumannii reference strains ATCC 17978 and ATCC 19606 in antimicrobial resistance mediated by the AdeABC efflux pump. Antimicrob. Agents Chemother. **65**, e0057021. (10.1128/AAC.00570-21) 34097477 PMC8284471

[50] Monk JM, Koza A, Campodonico MA, Machado D, Seoane JM, Palsson BO, Herrgård MJ, Feist AM. 2016 Multi-omics quantification of species variation of escherichia coli links molecular features with strain phenotypes. Cell Syst. **3**, 238–251. (10.1016/j.cels.2016.08.013) 27667363 PMC5058344

[51] Dewachter L, Fauvart M, Michiels J. 2019 Bacterial heterogeneity and antibiotic survival: understanding and combatting persistence and heteroresistance. Mol. Cell **76**, 255–267. (10.1016/j.molcel.2019.09.028) 31626749

[52] Dimitriu T, Matthews AC, Buckling A. 2021 Increased copy number couples the evolution of plasmid horizontal transmission and plasmid-encoded antibiotic resistance. Proc. Natl. Acad. Sci. U.S.A. **118**, e2107818118. (10.1073/pnas.2107818118) 34326267 PMC8346908

[53] Hernandez-Beltran JCR, Miró Pina V, Siri-Jégousse A, Palau S, Peña-Miller R, González Casanova A. 2022 Segregational instability of multicopy plasmids: a population genetics approach. Ecol. Evol. **12**, e9469. (10.1002/ece3.9469) 36479025 PMC9720003

[54] Kostylev M, Otwell AE, Richardson RE, Suzuki YJP. 2015 Cloning should be simple: Escherichia coli DH5α-mediated assembly of multiple DNA fragments with short end homologies. PLoS ONE **10**, e0137466. (10.1371/journal.pone.0137466) 26348330 PMC4562628

[55] Alam MK, Alhhazmi A, DeCoteau JF, Luo Y, Geyer C. 2016 RecA inhibitors potentiate antibiotic activity and block evolution of antibiotic resistance. Cell Chem. Biol. **23**, 381–391. (10.1016/j.chembiol.2016.02.010) 26991103

[56] Njenga R, Boele J, Öztürk Y, Koch HG. 2023 Coping with stress: how bacteria fine-tune protein synthesis and protein transport. J. Biol. Chem. **299**, 105163. (10.1016/j.jbc.2023.105163) 37586589 PMC10502375

[57] Léger L, Budin-Verneuil A, Cacaci M, Benachour A, Hartke A, Verneuil N. 2019 β-lactam exposure triggers reactive oxygen species formation in Enterococcus faecalis via the respiratory chain component DMK. Cell Rep. **29**, 2184–2191. (10.1016/j.celrep.2019.10.080) 31747593

[58] Gonzalez-Hunt CP, Wadhwa M, Sanders L. 2018 DNA damage by oxidative stress: measurement strategies for two genomes. Curr. Opin. Toxicol. **7**, 87–94. (10.1016/j.cotox.2017.11.001)

[59] Jones DL, Brewster RC, Phillips R. 2014 Promoter architecture dictates cell-to-cell variability in gene expression. Science **346**, 1533–1536. (10.1126/science.1255301) 25525251 PMC4388425

[60] Szmolka A, Nagy B. 2013 Multidrug resistant commensal Escherichia coli in animals and its impact for public health. Front. Microbiol. **4**, 258. (10.3389/fmicb.2013.00258) 24027562 PMC3759790

[61] Wang X *et al*. 2014 Heteroresistance at the single-cell level: adapting to antibiotic stress through a population-based strategy and growth-controlled interphenotypic coordination. MBio **5**, e00942–13. (10.1128/mBio.00942-13) 24520060 PMC3950525

[62] Falagas ME, Makris GC, Dimopoulos G, Matthaiou DK. 2008 Heteroresistance: a concern of increasing clinical significance? Clin. Microbiol. Infect. **14**, 101–104. (10.1111/j.1469-0691.2007.01912.x) 18093235

[63] Wistrand-Yuen E, Knopp M, Hjort K, Koskiniemi S, Berg OG, Andersson DI. 2018 Evolution of high-level resistance during low-level antibiotic exposure. Nat. Commun. **9**, 1599. (10.1038/s41467-018-04059-1) 29686259 PMC5913237

[64] Gullberg E, Cao S, Berg OG, Ilbäck C, Sandegren L, Hughes D, Andersson DI. 2011 Selection of resistant bacteria at very low antibiotic concentrations. PLoS Pathog. **7**, e1002158. (10.1371/journal.ppat.1002158) 21811410 PMC3141051

[65] Równicki M, Wojciechowska M, Wierzba AJ, Czarnecki J, Bartosik D, Gryko D, Trylska J. 2017 Vitamin B_12_ as a carrier of peptide nucleic acid (PNA) into bacterial cells. Sci. Rep. **7**, 7644. (10.1038/s41598-017-08032-8) 28794451 PMC5550456

[66] Kowalska-Krochmal B, Dudek-Wicher R. 2021 The minimum inhibitory concentration of antibiotics: methods, interpretation, clinical relevance. Pathogens **10**, 165. (10.3390/pathogens10020165) 33557078 PMC7913839

[67] Ruszczak A, Bartkova S, Zapotoczna M, Scheler O, Garstecki P. 2022 Droplet-based methods for tackling antimicrobial resistance. Curr. Opin. Biotechnol. **76**, 102755. (10.1016/j.copbio.2022.102755) 35841864

[68] Collins DJ, Neild A, deMello A, Liu AQ, Ai Y. 2015 The Poisson distribution and beyond: methods for microfluidic droplet production and single cell encapsulation. Lab Chip **15**, 3439–3459. (10.1039/c5lc00614g) 26226550

[69] van der Walt S, Schönberger JL, Nunez-Iglesias J, Boulogne F, Warner JD, Yager N, Gouillart E, Yu T, scikit-image contributors. 2014 scikit-image: image processing in Python. PeerJ **2**, e453. (10.7717/peerj.453) 25024921 PMC4081273

[70] Chorianopoulos NG, Lambert RJW, Skandamis PN, Evergetis ET, Haroutounian SA, Nychas GJE. 2006 A newly developed assay to study the minimum inhibitory concentration of Satureja spinosa essential oil. J. Appl. Microbiol. **100**, 778–786. (10.1111/j.1365-2672.2006.02827.x) 16553733

[71] Lambert RJ, Pearson J. 2000 Susceptibility testing: accurate and reproducible minimum inhibitory concentration (MIC) and non-inhibitory concentration (NIC) values. J. Appl. Microbiol. **88**, 784–790. (10.1046/j.1365-2672.2000.01017.x) 10792538

[72] Press WH, Teukolsky SA, Vetterling WT, Flannery BP. 2007 Numerical recipes, 3rd edn. New York: Cambridge University Press.

[73] Marquardt DW. 1963 An algorithm for least-squares estimation of nonlinear parameters. Journal of the Society for Industrial and Applied Mathematics **11**, 431–441. (10.1137/0111030)

[74] Foik I. 2024 Bacterial strain type and TEM-1 enzyme allele impact antibiotic susceptibility distribution in monoclonal populations: a single cell droplet approach (10.18150/MPGVXP) PMC12307058 40740715

[75] Shahryari S, Ahmad S, Foik IP, Jankowski P, Samborski A, Równicki M *et al*. 2025 Supplementary material from: Bacterial strain type and TEM-1 enzyme allele impact antibiotic susceptibility distribution in monoclonal populations: a single cell droplet approach. FigShare (10.6084/m9.figshare.c.7941526) PMC12307058 40740715

